# Assembly of Gold Nanorods on HSA Amyloid Fibrils to Develop a Conductive Nanoscaffold for Potential Biomedical and Biosensing Applications

**DOI:** 10.1038/s41598-018-26393-6

**Published:** 2018-06-19

**Authors:** Ramezan Ali Taheri, Yasin Akhtari, Tahereh Tohidi Moghadam, Bijan Ranjbar

**Affiliations:** 1 Nanobiotechnology Research Center, Baqiyatallah University of Medical Sciences, Tehran, Iran; 2 Department of Biomaterials Engineering, Faculty of High Technologies, Tarbiat Modares University, Tehran, Iran; 3 Department of Nanobiotechnology, Faculty of Biological Sciences, Tarbiat Modares University, Tehran, Iran; 4 Department of Biophysics, Faculty of Biological Sciences, Tarbiat Modares University, Tehran, Iran

## Abstract

Today, Gold Nanorods have promised variety of applications in conjugation with biomolecules of interest. Discovery of functional amyloids has also been highlighted with possible use in designing high performance materials. To exploit dual properties of both Nano and Bio counterparts in new functional materials, this effort has focused on synthesis of a potential hybrid system of Gold nanorods (GNRs) and HSA amyloid fibrils to develop a conductive nanoscaffold. UV-Vis spectroscopy, Thioflavin T (ThT) assay, Far-UV Circular Dichroism (CD) spectropolarimetry, fluorescence and Transmission Electron microscopy were used to characterize formation of the nanostructures and amyloid fibrils. Surface plasmon resonance of GNRs was also monitored upon interaction with HSA amyloid fibrils, showing that the plasmonic component of the hybrid system has maintained its characteristic rod morphology without any perturbations. Analysis of Nyquist plots for the hybrid nanoscaffold showed that the electronic behavior of the hybrid system has been enhanced due to the presence of the assembled GNRs. Results of this investigation highlight the possibility of fabricating hybrid nano-bioscaffolds as promising candidates in versatile biomedical and biosensing applications.

## Introduction

Gold nanoparticles have shown unique physicochemical properties for utilization in various medical applications such as diagnostics, imaging and therapies. Amongst gold nanoparticles of different morphologies, the rod shaped nanostructures are considered as a prime choice in many biomedical applications. Gold nanorods (GNRs) have shown outstanding properties over other nanoparticles, such as high stability, high absorption efficiency, tunable absorption in the biological window, and deep penetration of near-infrared light for destruction of tumor tissues^1,2^. The tiny nanostructures have the highest surface cross-section compared to other morphologies (i.e. cages and spherical), with higher efficiency in adhering to cells^3^. During the past decade, study on bioavailability, biocompatibility, self-assembly, functionalization and bioconjugation of GNRs with various biomolecules have led to design and fabrication of molecular nano probes for simultaneous detection of proteins^4–6^ and nucleic acids^7^ as well as development of molecular therapeutic agents^8^, controlled gene release^9,10^, imaging^11^, and hyperthermia^12^. Moreover, these nanostructures can easily be modified by thiol ended molecules that make them suitable for many different biological applications^13–15^. Therefore, interaction of these tiny nanostructures with biomolecules of interest promises simultaneous exploitation of both nano and bio features in form of hybrid systems for fruitful biomedical and biosensing applications.

Focusing on the biological component of hybrid systems, amyloid fibrils are misfolded structures of many proteins that are formed upon exposure to denaturing conditions. Nanoscale dimensions, chemical and mechanical strength, thermal stability and ease of functionalization are attractive features of amyloid fibrils^16^. Amyloid fibrils are considered to have been related with many neurodegenerative diseases including Parkinson’s disease, Huntington’s disease, type II diabetes, etc. Nonetheless, researches have proposed their brighter side with many positive functions recently, including their use as biomimetic materials which may act as bioscaffolds for cell support. Bhak *et al*. used α-synuclein fibrils as amyloid hydrogel for enzyme entrapment^17^. Herland *et al*. reported insulin amyloid fibrils conjugated with polymer APFO-12, as nanowires for optical applications^18^. Tao *et al*. developed silver nanocables using insulin amyloid fibrils with various functionalities and potential applications in conductive materials, sensors and catalysts^19^. Moreover, amyloid fibrils have shown fruitful applications in tissue engineering. Horii *et al*. reported that peptide scaffolds promote proliferation and osteogenic differentiation of mouse MC3T3-E1 cells^20^. Reeba *et al*. designed and fabricated novel self-healing hydrogels composed of amyloid fibril scaffolds for mesenchymal stem cells differentiation, utilizing the small size, ease of custom synthesis and thixotropic nature of amyloid-based hydrogels^21^.

Taking the promising applications of both gold nanorods and amyloid fibrils into account, in this effort fabrication of a hybrid scaffold consisting of plasmonic GNRs and HSA amyloid fibrils has been attempted in view of assembly of the nanostructures on the biological template to dictate conductivity of GNRs to a mechanically and chemically strong scaffold. Each components of the hybrid scaffold has been characterized via spectroscopic and microscopic techniques. To the best of our knowledge, assembly of gold nanorods on HSA amyloid fibrils to fabricate a conductive nanoscaffold has not been reported so far.

## Results and Discussion

### Characterizations of Gold Nanorods

**Figure 1:**
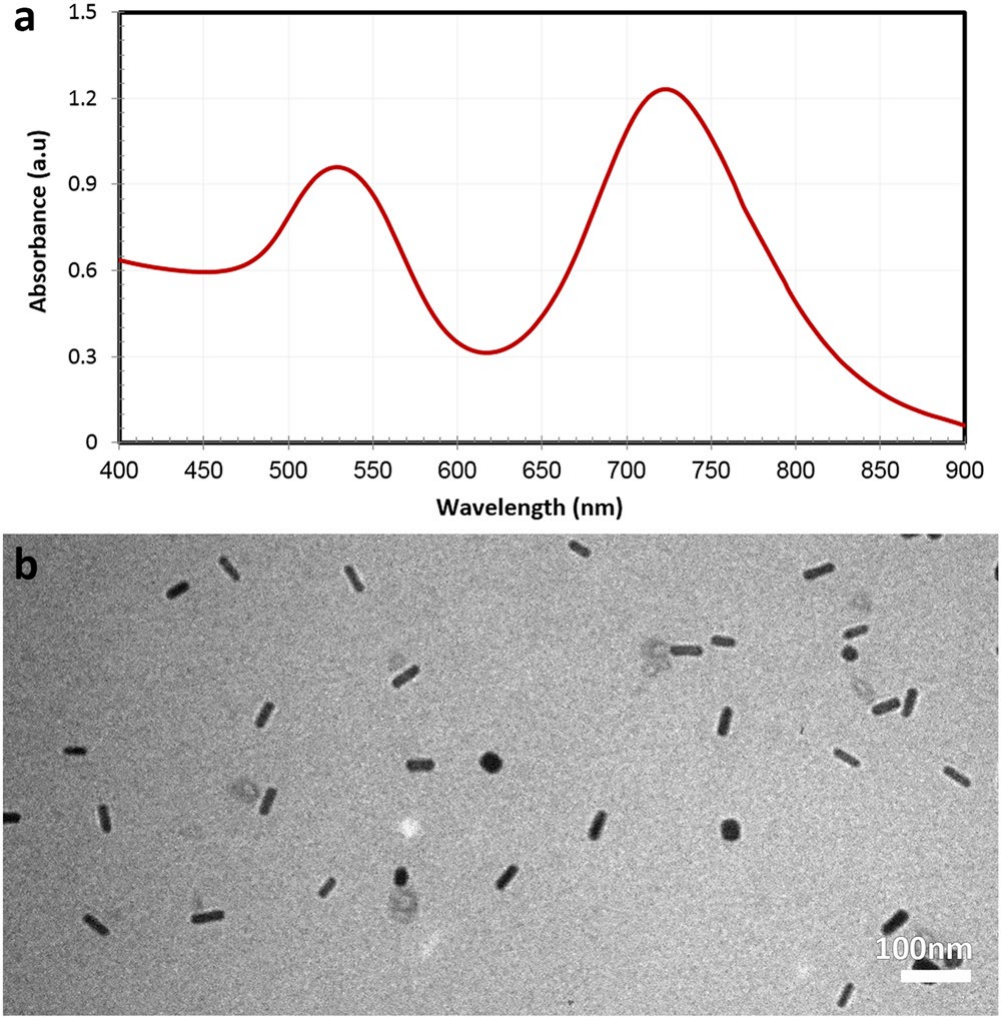
(**a**) Characteristic SPR band of GNRs and (**b**) TEM image of Gold Nanorods.

Formation of gold nanoparticles with rod morphology was monitored by appearance of two absorption bands in the visible and near infrared region (400–900 nm). The two surface plasmon resonance (SPR) bands of gold nanorods in this region represent oscillation of electrons in the conduction band of the nanostructures along the short and long axis (Fig. 1a). The weaker band at 525 nm indicates transverse SPR of gold nanorods; whereas the stronger band at 725 nm represents longitudinal surface plasmon resonance (LSPR) of the nanostructures. It is worth to mention that the wavelength region where the LSPR appears is dependent upon the aspect ratio and synthesis conditions of GNRs. Therefore, any variation in the concentration of reactants will affect the position of SPR bands as well as their intensities. Various aspect ratios of GNRs are suitable for typical applications. For example, longer gold nanorods can be utilized in imaging purpose; whereas shorter ones are prefect alternatives for photothermal therapy applications.

Transmission electron microscopy image of the purified samples also confirmed formation of nanostructures with rod morphology (Fig. 1b). The mean aspect ratio of gold nanorods was estimated to be 3.7 ± 0.52, with average dimension of 37 nm in length and 10 nm in width.

### Characterizations of HSA Amyloid Fibrils

#### Thioflavin-T Assay

**Figure 2:**
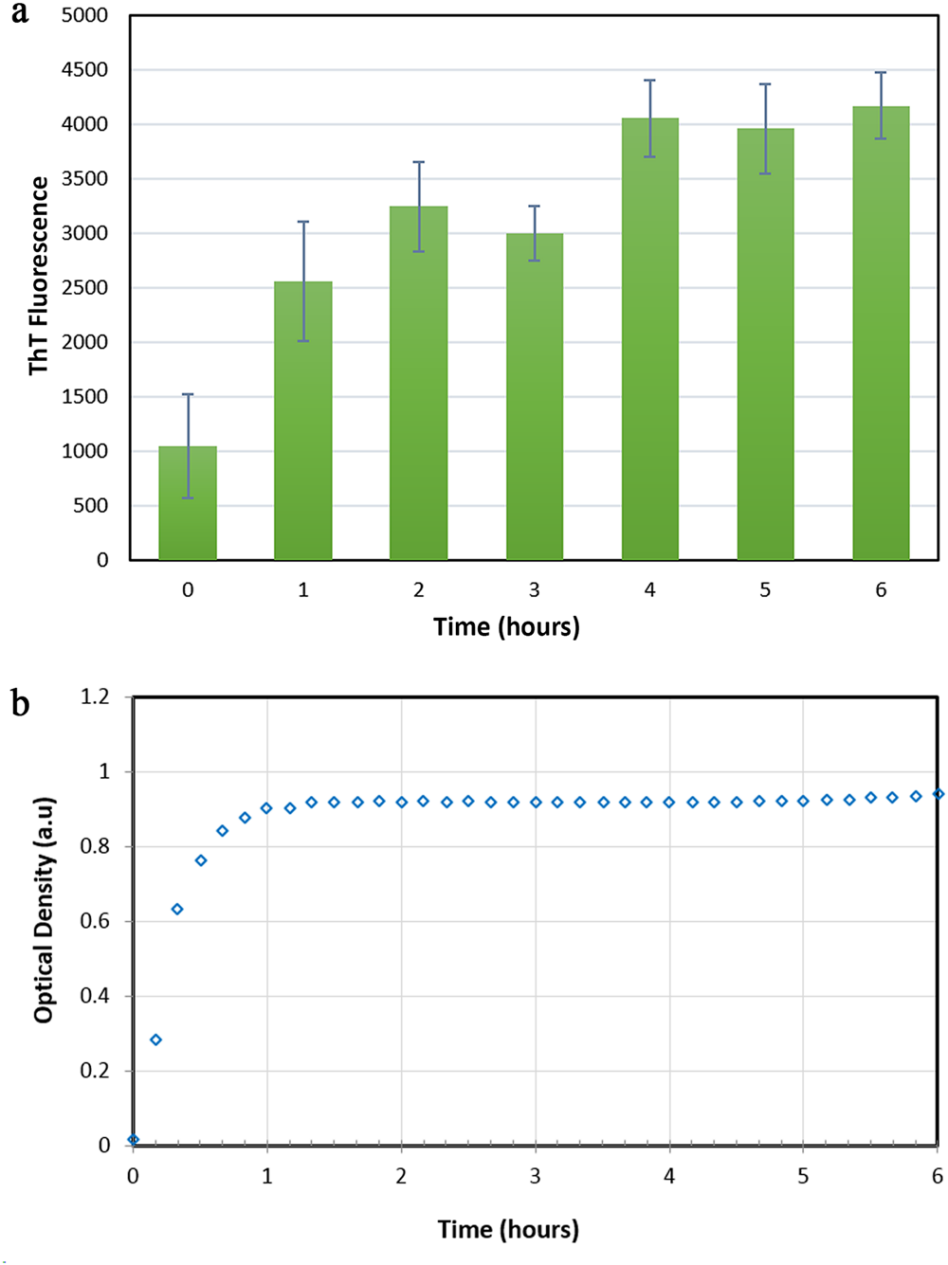
(**a**) ThT emission of HSA incubation during 6 hours. (**b**) Optical Density of HSA Amyloid Fibril formation at 400 nm.

Thioflavin-T (ThT) is a common histological dye used to monitor formation of beta-structures in proteins. The dye specifically interacts with cross-β structure and exhibits secondary structural transformations into β-structure. Measurement of ThT fluorescence can be considered as a useful method to investigate the rate of amyloid aggregation formation for proteins. As shown in Fig. 2a, ThT fluorescence spectroscopy of HSA was monitored during 6 hours. The ascending trend in the fluorescence intensity for the first half time interval represented increasing in β-sheet structure and formation of amyloid fibrils. Within the second half interval of the procedure, a constant rate of ThT fluorescence was observed, representing completion of amyloid fibrils formation.

#### Turbidity Test of HSA Aggregation Process

Measuring of protein absorbance in the visible wavelength (400 nm) can indirectly indicate protein aggregation. Increase in the turbidity of protein solution is followed by increase in the absorbance intensity. Figure 2b shows optical density of HSA solution upon fibrillation process in 6 hours. In the first hour of the process, HSA began to form aggregated structures with turbid appearance. After one hour, the intensity remained constant and reached to plateau. This indicates end of aggregation kinetics and conformational changes in protein structure. It should be emphasized that turbidity measurement of protein is an inconclusive method to monitor protein aggregations states, and cannot confirm formation of amyloid fibrils. Therefore, it is necessary to exploit other supplementary tests (ThT and CD) to assure specific conformational changes.

#### Fluorescence Microscopy Images of Amyloid Fibrils

**Figure 3:**
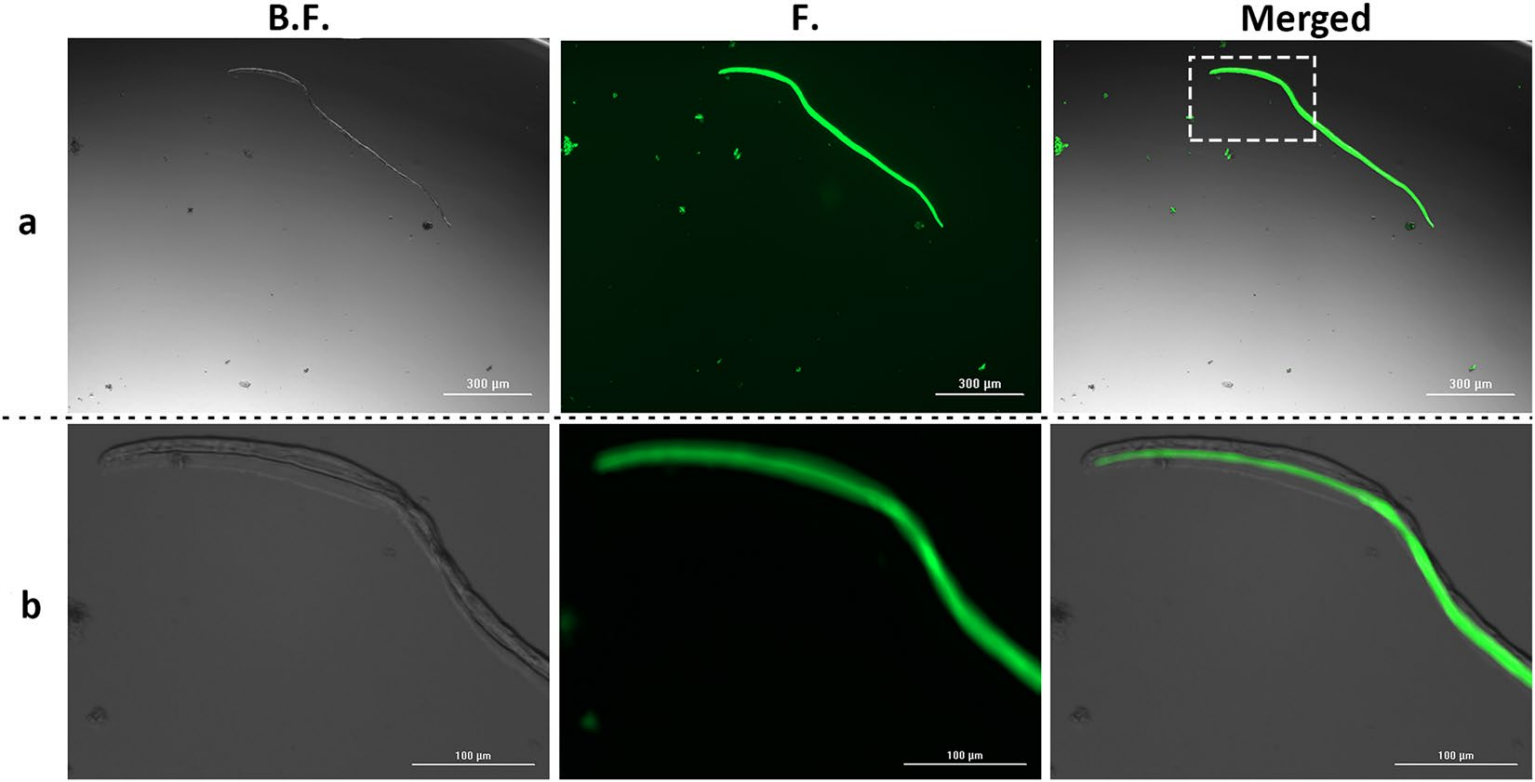
(**a**) ThT stained fluorescence microscopy images of HSA amyloid fibrils from the same area under bright field (B.F.), fluorescence (F.) and merged mode. (**b**) 5 × magnification view of the dashed white box.

Figure 3 shows fluorescence microscopic images of HSA amyloid fibrils stained with ThT. The merged images represented in this figure (integrated from the bright field and fluorescence mode) show that fluorescence is only observed from the fibrils^22^.

#### Far-UV CD Spectroscopy of HSA fibrillation

**Figure 4:**
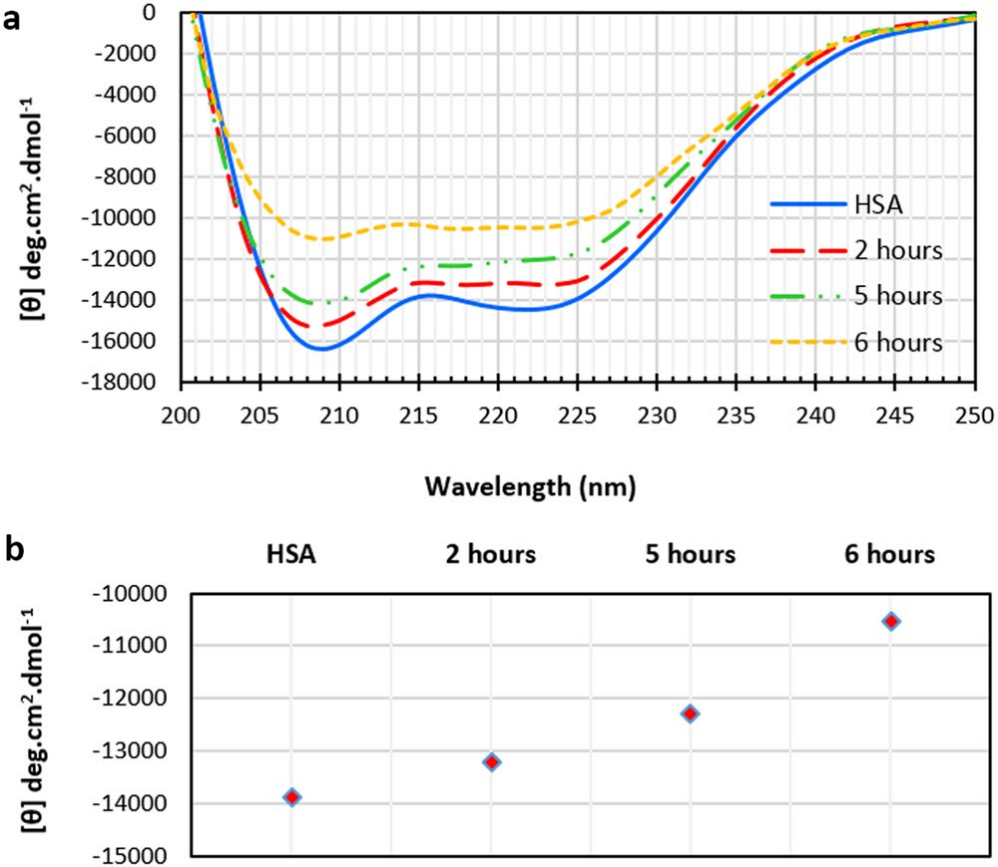
(**a**) Kinetic of CD spectra of HSA sample at 65 °C. (**b**) Time evolution of CD signal measured at 222 nm at 65 °C.

Circular Dichroism spectropolarimetry is a useful technique for monitoring secondary structure of proteins. Conformational changes of biomolecules is given by difference in the absorbance of left and right circularly polarized light in the UV region^23^. Figure 4a shows Far-UV CD spectra of human serum albumin at different times of incubation under amyloid fibril formation conditions. Data was smoothed and analyzed by Jasco secondary structural analysis software. As depicted in the Figure, HSA CD spectra have two typical characteristic bands of α-helix at 208 and 222 nm. After incubation for 2 hours, conformation of the biomolecule has experienced alterations in its secondary structure. Figure 4b shows changes in the band intensity at 222 nm. A glance at intensity changes at 208 and 222 nm indicates decrease in the helical content of HSA upon formation of amyloid fibril structures. Further incubation showed appearance of a shoulder at 217 nm, representing an increase of β-sheet content in the biomolecule’s structure. Hence, after 6 hours of incubation at 65 °C, HSA acquires structural changes and transformation into amyloid fibrils.

#### TEM image of HSA amyloid fibrils

**Figure 5:**
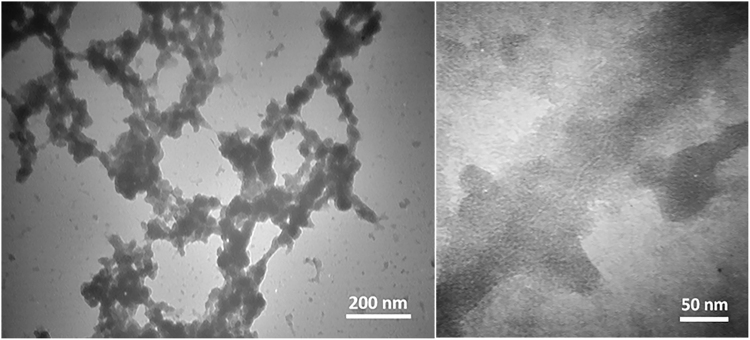
TEM images of HSA amyloid fibrils.

Transmission electron microscopy image of HSA solution after 6 hours of incubation at 65 °C in 60% (v/v) ethanol showed formation of amyloid fibril aggregates (Fig. 5). Since the sample was negatively stained with uranyl acetate to obtain appropriate contrast, amyloid fibrils are observed as dark threadlike fibrillar species clumped together. The dense fibrillar networks of HSA were estimated to have diameters of 70–90 nm. It would be mention worthy that various parameters such as pH, temperature, incubation time, ionic strength etc., are considered as key factors in formation and quality of amyloid fibril structures^24^.

### Interaction of GNRs with HSA Amyloid Fibrils

**Figure 6:**
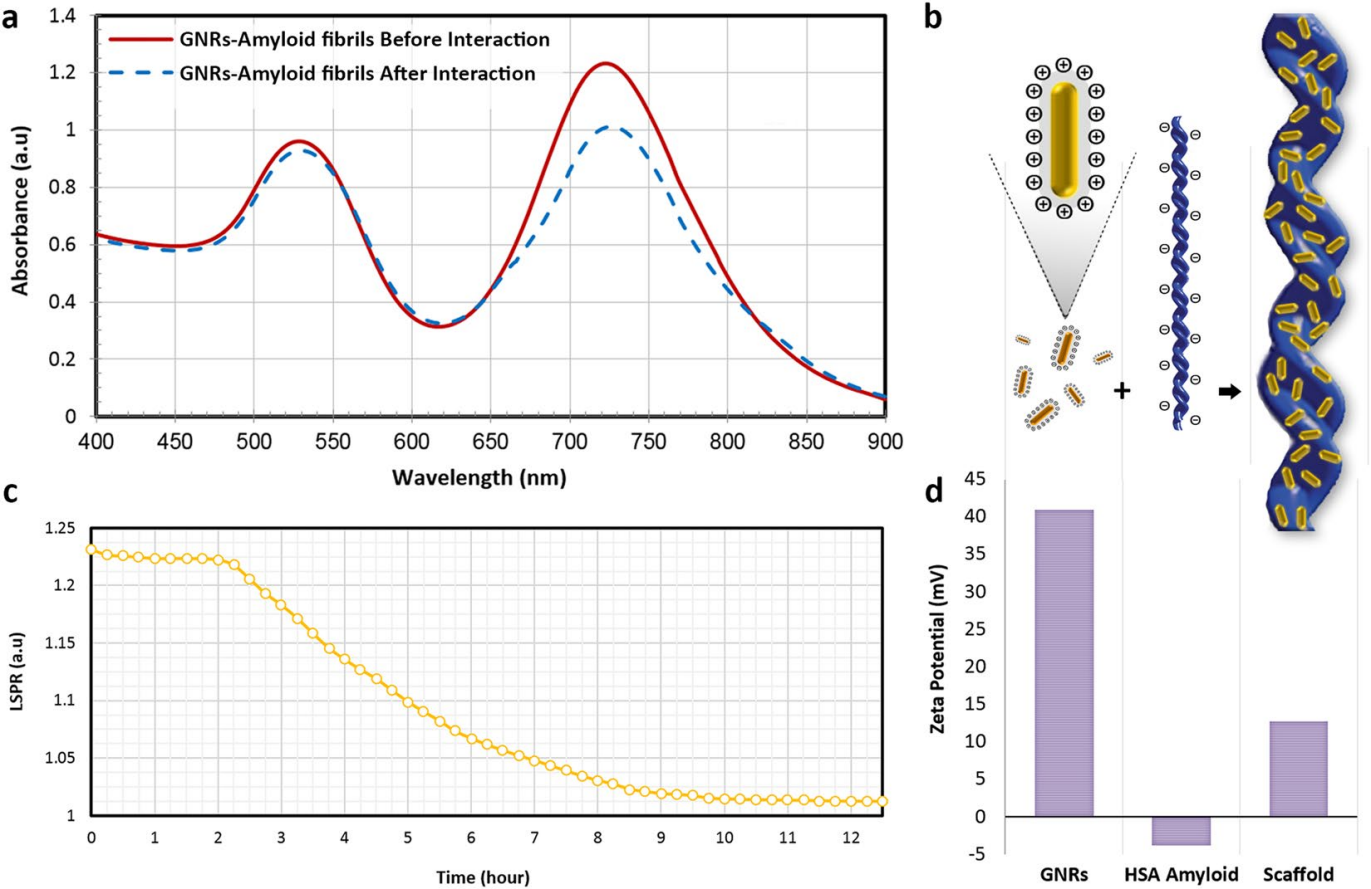
(**a**) SPR spectra of GNRs before and after interaction with HSA amyloid fibrils. (**b**) Schematic representation of assembly of GNRs on HSA amyloid fibrils. (**c**) Changes in intensity of LSPR of GNRs during 12 hours of interaction with HSA amyloid fibrils. (**d**) Zeta potential measurements of the nanoscaffold before and after assembly of GNRs.

Figure 6a compares the characteristic SPR bands of GNRs before and after interaction with HSA amyloid fibrils after 12 hours. As anticipated, intensity of both SPR bands decreased, representing interaction of the nanostructures with amyloid fibrils. However, a glance at Fig. 6a shows that longitudinal SPR of GNRs (LSPR) experienced more change with respect to their transverse (TSPR) band. This phenomenon usually occurs due to the high sensitivity of the oscillating conduction electrons of the GNRs on the long axis to trace changes in the local environment which, in turn, makes such tiny particles promising candidates in design of molecular nanoprobes for diagnostic applications. Schematic representation of GNRs upon interaction with HSA amyloid fibrils has been depicted Fig. 6b.

To monitor the stability of nanostructures on the biotemplate, characteristic SPR bands of GNRs were recorded upon interaction with HSA amyloid fibrils. Figure 6c represents changes in the intensity of longitudinal surface plasmon resonance (LSPR) band upon interaction with amyloid fibril structures. Within 2 hours of incubation at the ambient temperature, there was negligible change in the LSPR intensity; whereas it decreased to some extent in the following 7 hours. Further incubation of GNRs with the biotemplate showed that the interaction between GNRs and amyloid fibrils reached to an equilibrium state and did not experience intensity changes. Hence, taking the shape of the SPR bands (Fig. 6a) and their intensity changes (Fig. 6c) into account, the HSA biotemplate did not induce remarkable perturbations in the structure and typical rod morphology of GNRs. This is a very important issue for the case of plasmonic nanostructures such as gold and silver which are very sensitive to trace changes in the refractive index for nanobiosensing applications. Notable decrease in the intensity and change in the overall appearance of LSPR band of these nanostructures can clearly be considered as an indicator of reversible aggregation, total change of rod morphology, leading to loss of the expected outstanding plasmonic and conductive properties.

Figure 6d depicts analysis of zeta potential of the samples before and after formation of the hybrid scaffold. Based on the difference in the surface charge of the nanostructures and the biological template, GNRs can electrostatically interact with HSA amyloid fibrils. Gold nanorods contain zeta potential of +41 mV due to the presence of cationic surfactant layers around their matrix; whereas HSA amyloid fibrils show a net negative charge with pH above the isoelectric point of the protein (−3.8 mV). The hybrid system showed a zeta potential value of +12.8 mV, representing interaction of the nanostructures with the biological template.

**Figure 7:**
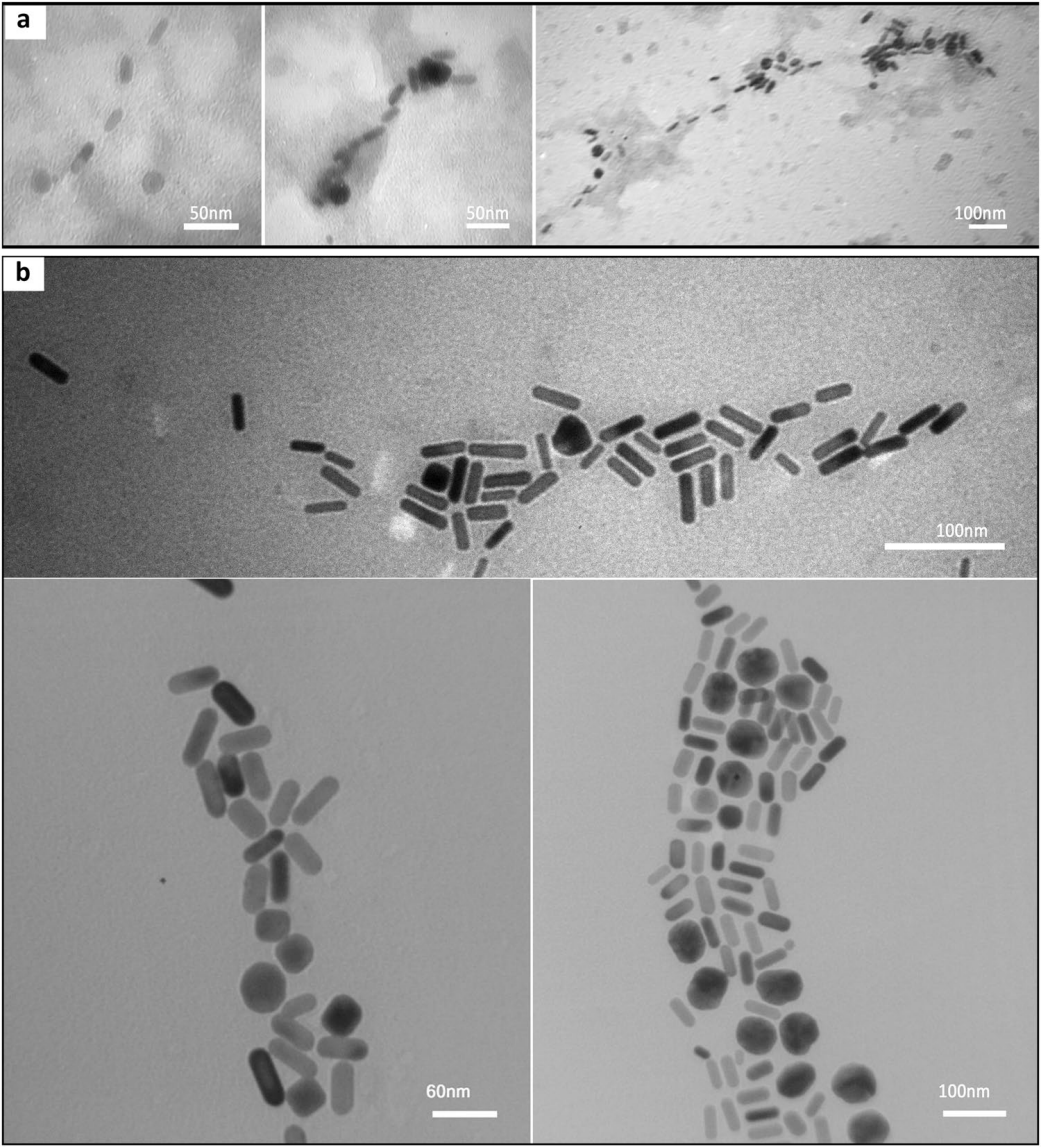
TEM images of hybrid nanoscaffold of GNRs and HSA amyloid fibrils (**a**) with uranyl acetate negative staining, and (**b**) without staining.

Figure 7 depicts transmission electron microscopy images of the hybrid scaffold containing both the plasmonic nanostructures and the biological template. A glance at microscopy images shows that GNRs have anchored to HSA amyloid fibrils. Comparison of TEM image of bare GNRs (Fig. 1b) with their hybrid counterpart indicates that the nanostructures have been directed on the matrix of fibrils in an assembled fashion. Furthermore, in consistency with SPR spectra (Fig. 6a), TEM images of the hybrid system mostly represents the intact rod-shaped morphology of the nanostructures. Nevertheless, prior to microscopy characterization, some of the nanostructures have lost their typical rod morphology on the biotemplate due to the aging phenomena, leading to decrease in the yield of the interacted GNRs with respect to the bare nanostructures.

### Electrical Behavior of Hybrid Scaffold

**Figure 8:**
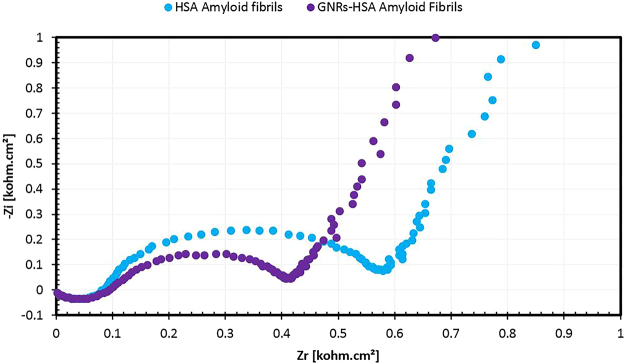
Nyquist plots for electrochemical impedance spectroscopy (EIS) comparison of HSA amyloid fibrils and hybrid nanoscaffolds.

Considering the potential application of our hybrid system in developing conductive nanoscaffolds, electrical properties of HSA amyloid fibrils and hybrid scaffold solutions were tested by electrochemical impedance spectroscopy (EIS). EIS is a technique in which a voltage sinusoidal wave is applied with known amplitude over a frequency range^25^. Figure 8 shows Nyquist plots as a result of EIS, in which, (Zr) is real impedance and (−Zi) is imaginary impedance, with data shown as a semicircle. EIS spectra corresponding to each sample can be seen in this figure. Upon assembly of GNRs on the biological template, change in the EIS spectrum was noticed in comparison with bare amyloid fibril structures. Decrease in diameter of the semicircle can be related to reduction of electron transfer resistance at the electrode surface. Therefore, observing a semicircle of smaller diameter for the hybrid nanoscaffold system with respect to the bare HSA amyloid fibrils suggests that the electronic behavior of the overall system has been enhanced due to the assembly of the rod-shaped gold nanostructures. This, in turn, promises the possibility of developing conductive nanoscaffolds both for variety of biological and non-biological applications.

In the context of the real application for the hybrid nano-bio system reported in this study, there are no traces of utilizing amyloid fibrils and gold nanorods in tissue/neural engineering and the idea is still in its infancy. Nevertheless, there are some reports in the recent years, which have focused on introducing biomedical applications of other types of scaffolds in conjugation with inorganic nanoparticles such as silver and gold. Interestingly, for the first time, Tal Devir *et al*. showed that nanocomposites of inorganic nanomaterials in polymeric matrices are likely to be used to enhance the structure, phenotype and function of engineered cardiac tissue. The key limitation of porous matrices that are currently utilized for cardiac tissue engineering is that their pore walls limit cell–cell interaction and delay electrical signal propagation. Since inorganic nanostructures can interact with cardiomyocytes and neurons to create electronic interfaces, Tal Devir *et al*. developed three-dimensional nanocomposites of gold nanowires within macroporous scaffolds to investigate the electrical communication throughout the cell-seeded scaffold and organization of the functioning tissue. In this study, they showed how incorporating gold nanowires within alginate scaffolds successfully bridged the electrically resistant pore walls of alginate and improved electrical signal propagation between the adjacent cardiac cells and throughout the scaffold. Tissues grown on these composite matrices were thicker and better aligned than those grown on pristine alginate, and the nanowires imparted phenotypic traits consistent with enhanced electrical and mechanical coupling and contractile properties. Furthermore, higher levels of the proteins involved in muscle contraction and electrical coupling were detected in the composite matrices. Therefore, the integration of conducting nanowires within three-dimensional scaffolds is expected to improve the therapeutic value of current cardiac patches, creating more homogeneous patches with stronger contractile properties that could be implanted on the affected surface of a heart after Myocardial infarction (MI)^26^.

In another example, Cohen-Karni *et al*. reported that surface chemistry modification of biomaterials by gold nanoparticles can induce cell proliferation and differentiation. They used silk nanofibers as a scaffold due to their desirable mechanical and biological properties. In order to improve cell-biomaterial interactions, gold nanoparticles were dispersed throughout the silk nanofibers. Results showed that Young’s modulus of the silk fibers remarkably increased by 70% after incorporation of nanoparticles as a sign of enhanced mechanical properties. Culturing human mesenchymal stem cells on the gold nanoparticle-incorporated fibers showed larger cell area compared to their bare counterpart as well as increased cell size and density, indicating enhancement of cellular spreading. Increase in the surface roughness of silk fibers by gold nanoparticles in this research presented that such modifications can affect a wide range of cell behaviors in tissue engineering applications^27^.

Quite recently, there have been a number of reports focusing on conductivity of GNRs in different systems. Zhu *et al*. studied on GNRs-incorporated gelatin methacryloyl based bioink in order to develop printing 3D functional cardiac tissue constructs. It was reported that GNRs improved electrical propagation between cardiac cells. Furthermore, GNRs based bioink could also assist in regenerating other electrogenic tissues^28^.

Navaei *et al*. fabricated conductive biomaterial for cardiac tissue engineering. In their report, GNRs were exploited to enhance electrical conductivity of biomaterial by incorporating them into gelatin methacrylate (GelMA) hybrid hydrogels. The hybrid scaffold developed by Navaei *et al*. showed superior electrical and mechanical properties. Furthermore, high cell retention, viability, metabolic activity and facilitated cell-cell signaling as well as electrical signal propagation can be considered as other outcomes of such hybrid constructs, in which, the nanostructures are shown to have played a key role in guiding higher cell adhesion and retention. Excellent characteristics of nanoengineered GelMA-GNRs hybrid hydrogel introduce them as promising candidates in cardiac tissue engineering applications^29^.

Taking the recent reports into account, developing hybrid systems could be generalized to other biomaterials such as fibers, or amyloid fibrils of strong mechanical and chemical resistance (in this study) which can be simultaneously utilized with inorganic nanoparticles of outstanding characteristics (gold nanorods) in diseases of conductive tissues. Engineering cardiac patches for treating damaged heart tissues after a heart attack or developing neural networks of higher biocompatibility and signal transfer efficiency in neuroscience and neural engineering can be highlighted as the promising biomedical applications of such hybrid nanoscaffolds. Moreover, taking the advantage of outstanding surface plasmon resonance properties of GNRs and their extreme sensitivity to trace changes in their local environment, a hybrid system consisting of such tiny rod-shaped nanostructures and amyloid fibrils might pave the way for developing a new biosensing strategy for the upcoming generation of plasmonic nanobiosensors and early diagnosis of pathogens and cancers^30^.

## Conclusion

This effort has focused on synthesis of a hybrid nanoscaffold, exploiting the outstanding properties of rod-shaped gold nanostructures and HSA amyloid fibrils, in view of assembling GNRs on a mechanically and chemically stable biological template. The nanoscaffold was characterized via various spectroscopic and microscopy techniques including Thioflavin-T Fluorescence, Circular Dichroism Spectropolarimetry, UV-Visible, Electrochemical Impedance Spectroscopy, Dynamic Light Scattering, Fluorescence and Transmission Electron Microscopy. Upon interaction with HSA amyloid fibrils, the nanostructures anchored to the biological template in an assembled fashion, while maintaining their characteristic plasmonic property without any perturbations in the structure and morphology. Analysis of Nyquist plots for the hybrid nanoscaffold showed that the electronic behavior of the hybrid system has been enhanced due to the presence of the assembled GNRs. Results of this investigation encourage design and development of a new generation of hybrid nanoscaffolds for fruitful applications in tissue engineering and nanobiosensing purposes.

## Methods

### Chemicals

Hydrogen tetrachloroaurate (III), Human Serum Albumin, Sodium Borohydride, Silver nitrate, Cetyltrimethylammonium bromide (CTAB), Acid Ascorbic, Hydrogen chloride, Thioflavin-T (ThT), and Sodium hydroxide were procured from.

### Synthesis of Gold Nanorods

Gold Nanorods were synthesized according to seed mediated growth^31,32^, as modified by Tohidi Moghadam *et al*.^2,33^. Briefly, seed solution was prepared by addition of HAuCl_4_. 3H_2_O (250 μL, 0.01 M) to CTAB (7.5 mL, 0.095 M) and followed by immediate addition of ice-cold NaBH_4_ solution (600 μL, 0.01 M). The reactants were mixed by rapid inversion for 2 minutes and kept undisturbed for a minimum of 2 hours at room temperature. The growth solution was then prepared by sequential addition of AgNO_3_ (60 μL, 0.01 M), ascorbic acid (64 μL 0.10 M) in mixture of HAuCl_4_. 3H_2_O (400 μL, 0.01 M) and CTAB (9.5 mL, 0.095 M). Finally, 40 μL of seed solution was added and rod-shaped nanostructures were formed after several hours^2^.

To remove excess cationic surfactant (CTAB) from gold nanorods, the solution was centrifuged twice at 13500 rpm for 10 min, and the precipitate was diluted by distilled water. The solution was then sonicated for several minutes to disperse the nanorods.

### Characterization of Gold Nanorods

To characterize gold nanorods, surface plasmon resonance of GNRs was monitored in the wavelength region of 400–900 nm, using UV-Vis PerkinElmer Lambda 25 Spectrometer. Size, morphology and distribution of gold nanoparticles were characterized by transmission electron microscopy (TE 2000 Ziess electron microscope) via deposition of GNRs on carbon coated copper grids.

### Preparation of Human Serum Albumin Amyloid Fibrils

10 mg.ml^−1^ HSA was dissolved in freshly prepared 60% (v/v) Ethanol and pH was adjusted to 7.4 by NaOH. Samples were then incubated in water-bath at 65 °C for 6 hours. Sampling was carried out each 30 minutes.

### Far-UV Circular Dichroism Spectroscopy

Circular Dichroism (CD) measurements were carried out on a JASCO spectropolarimeter (J-715) to monitor secondary structural changes in protein’s structure. The instrument was calibrated with D-10-camphorsulphonic acid. The protein sample was diluted to a final concentration of 200 μg.ml^−1^ in a quartz cuvette. Far ultra-violet (UV) CD region was scanned between 200 nm and 250 nm with a bandwidth of 1 nm. All spectra were recorded at ambient temperature.

Results were recorded in terms of molar ellipticity [θ], in *deg. cm*^2^*.dmol*^*−1*^, as depicted from the following equation:

1 $$[{\rm{\theta }}]=100[{\rm{\theta }}]{\rm{Mw}}/{\rm{c}}{\rm{.l}}{\rm{.n}}$$

where, *θ* is the measured ellipticity in degrees, *c* is the protein concentration in mg.ml^−1^, *l* is the path length in cm, *Mw* is the molecular weight of the protein and *n* is the number of amino acid residues. Data was smoothed and analyzed by Jasco software, after subtracting the buffer contribution from the original protein spectrum^2^.

### Turbidity test of amyloid fibril formation

To obtain amyloid fibril structures of HSA, 10 mg.ml^−1^ HSA solution was incubated at 65 °C, using Peltier accessory coupled to UV-Vis PerkinElmer Lambda25 spectrophotometer. Absorbance was recorded at 400 nm during the fibrillation process.

### Thioflavin-T (ThT) Assay

A stock solution of ThT was prepared (250 μM) in double distilled water, and protected from light to prevent quenching. For *in-situ* ThT fluorescence measurements, 20 μM ThT was added to freshly prepared solutions of HSA (10 mg.ml^−1^, 60% v/v ethanol, pH 7.4) in 96-wells polystyrene. Three replicates were done for each sample to minimize the well-to-well variation. Fluorescence was measured by Cytation3 Cell Imaging Multi-Mode Reader (BIOTEK INSTRUMENT, WINOOSKI, VT). The excitation and emission wavelengths were 440 nm and 485 nm, respectively.

For fluorescence microscopy analysis of HSA amyloid fibrils, 10 microliters of 1 mM ThT was mixed with 90 μL of HSA amyloid fibrils. Samples were placed on a glass slide covered with a coverslip and monitored by Cytation3 Cell Imaging Multi-Mode Reader^24^.

### Transmission electron microscopy of HSA amyloid fibrils

For Transmission electron microscopy images, 20 *µ*L of the 50-fold diluted sample was placed on a Formvar Carbon film coated on 300 mesh copper grid (EMS) for 2 min. Excess liquid was absorbed with filter paper, and the sample was negatively stained with 20 *µ*L 2% uranyl acetate for 1–2 min. The grid was allowed to air dry, and examined at an accelerating voltage of 100 kV.

### Interaction of GNRs with HSA Amyloid Fibrils

A sample containing 2 ml purified GNRs was sonicated and added to 0.5 ml HSA amyloid fibrils solution (10 mg.ml^−1^ HSA). The mixture was mildly shaken overnight.

### Electrochemical Impedance Spectroscopy

Electrochemical impedance spectroscopy (EIS) measurements of HSA amyloid fibrils and hybrid scaffold were performed using OrigaLys impedance meter (France) with frequency range between 1 Hz and 1 MHz and AC voltage of 700 mV, generating a Nyquist plot. Each solution was placed into an electrochemical cell and EIS measurements were performed with three-electrode system, consisting of platinum as working and auxiliary electrodes and Ag/AgCl as the reference electrode. All experiments were performed at room temperature.

## References

[1] Huang X, Neretina S, El-Sayed MA (2009). Gold nanorods: from synthesis and properties to biological and biomedical applications. Advanced materials.

[2] Tohidi Moghadam T, Ranjbar B, Khajeh K (2012). Conformation and activity of lysozyme on binding to two types of gold nanorods: a comparative study. International journal of biological macromolecules.

[3] Gong N (2015). Effects of the physicochemical properties of gold nanostructures on cellular internalization. Regenerative Biomaterials.

[4] Niidome T (2006). PEG-modified gold nanorods with a stealth character for *in vivo* applications. J Control Release.

[5] Yu C, Irudayaraj J (2007). Multiplex Biosensor Using Gold Nanorods. Analytical chemistry.

[6] Mohseni S, Moghadam TT, Dabirmanesh B, Jabbari S, Khajeh K (2016). Development of a label-free SPR sensor for detection of matrixmetalloproteinase-9 by antibody immobilization on carboxymethyldextran chip. Biosensors and Bioelectronics.

[7] Ma Z, Tian L, Wang T, Wang C (2010). Optical DNA detection based on gold nanorods aggregation. Analytica chimica acta.

[8] Li Z (2009). RGD-conjugated dendrimer-modified gold nanorods for *in vivo* tumor targeting and photothermal therapy. Molecular pharmaceutics.

[9] Takahashi, H., Niidome, Y. & Yamada, S. Controlled release of plasmid DNA from gold nanorods induced by pulsed near-infrared light. *Chemical communications*, 2247–2249, 10.1039/b500337g (2005). 10.1039/b500337g 15856111

[10] Chen CC (2006). DNA-gold nanorod conjugates for remote control of localized gene expression by near infrared irradiation. Journal of the American Chemical Society.

[11] He W (2008). Two-photon Luminescence Imaging of Bacillus Spores Using Peptide-functionalized Gold Nanorods. Nano research.

[12] Huang H-C, Rege K, Heys JJ (2010). Spatiotemporal temperature distribution and cancer cell death in response to extracellular hyperthermia induced by gold nanorods. ACS nano.

[13] Taheri, R. A., Rezayan, A. H., Rahimi, F., Mohammadnejad, J. & Kamali, M. Development of an immunosensor using oriented immobilized anti-OmpW for sensitive detection of Vibrio cholerae by surface plasmon resonance. *Biosens. Bioelectron*. **86**, 484–488, 10.1016/j.bios.2016.07.006 (2016). 10.1016/j.bios.2016.07.006 27442077

[14] Taheri, R. A., Rezayan, A. H., Rahimi, F., Mohammadnejad, J. & Kamali, M. Comparison of antibody immobilization strategies in detection of Vibrio cholerae by surface plasmon resonance. *Biointerphases* **11**, 041006, 10.1116/1.4971270 (2016). 10.1116/1.4971270 27923270

[15] Taheri, R. A., Rezayan, A. H., Rahimi, F., Mohammadnejad, J. & Kamali, M. Evaluating the potential of an antibody against recombinant OmpW antigen in detection of Vibrio cholerae by surface plasmon resonance (SPR) biosensor. *Plasmonics* **12**, 1493–1504, 10.1007/s11468-016-0411-2 (2017).

[16] Mankar, S., Anoop, A., Sen, S. & Maji, S. K. Nanomaterials: amyloids reflect their brighter side. *Nano reviews* **2**, 10.3402/nano.v2i0.6032 (2011). 10.3402/nano.v2i0.6032 PMC3215191 22110868

[17] Bhak G, Lee S, Park JW, Cho S, Paik SR (2010). Amyloid hydrogel derived from curly protein fibrils of alpha-synuclein. Biomaterials.

[18] Herland A, Thomsson D, Mirzov O, Scheblykin IG, Inganas O (2008). Decoration of amyloid fibrils with luminescent conjugated polymers. Journal of Materials Chemistry.

[19] Tao L, Gao Y, Wu P, Lu X, Gao F (2015). Insulin templated synthesis of single-crystalline silver nanocables with ultrathin Ag cores. RSC Advances.

[20] Horii A, Wang X, Gelain F, Zhang S (2007). Biological designer self-assembling peptide nanofiber scaffolds significantly enhance osteoblast proliferation, differentiation and 3-D migration. PloS one.

[21] Jacob RS (2015). Self healing hydrogels composed of amyloid nano fibrils for cell culture and stem cell differentiation. Biomaterials.

[22] Pradhan N, Jana D, Ghorai BK, Jana NR (2015). Detection and Monitoring of Amyloid Fibrillation Using a Fluorescence “Switch-On” Probe. ACS applied materials & interfaces.

[23] Ranjbar B, Gill P (2009). Circular dichroism techniques: biomolecular and nanostructural analyses- a review. Chemical biology & drug design.

[24] Pandey NK, Ghosh S, Dasgupta S (2010). Fibrillation in human serum albumin is enhanced in the presence of copper(II). The journal of physical chemistry. B.

[25] Scott DW, Alseiha Y (2017). Determining detection limits of aqueous anions using electrochemical impedance spectroscopy. Journal of Analytical Science and Technology.

[26] Dvir T (2011). Nanowired three dimensional cardiac patches. Nature nanotechnology.

[27] Cohen-Karni T (2012). Nanocomposite Gold-Silk Nanofibers. Nano letters.

[28] Zhu K (2017). Gold Nanocomposite Bioink for Printing 3D Cardiac Constructs. Advanced Functional Materials.

[29] Navaei A (2016). Gold nanorod-incorporated gelatin-based conductive hydrogels for engineering cardiac tissue constructs. Acta biomaterialia.

[30] Tohidi Moghadam T, Ranjbar B (2015). Heat induced aggregation of gold nanorods for rapid visual detection of lysozyme. Talanta.

[31] Nikoobakht B, El-Sayed MA (2003). Preparation and Growth Mechanism of Gold Nanorods (NRs) Using Seed-Mediated Growth Method. Chemistry of Materials.

[32] Gole A, Murphy CJ (2004). Seed-Mediated Synthesis of Gold Nanorods: Role of the Size and Nature of the Seed. Chemistry of Materials.

[33] Tohidi Moghadam T (2011). Interaction of lysozyme with gold nanorods: conformation and activity investigations. International journal of biological macromolecules.

